# One size does not fit all: a support vector machine exploration of multiclass cognitive state classifications using physiological measures

**DOI:** 10.3389/fnrgo.2025.1566431

**Published:** 2025-06-18

**Authors:** Jonathan Vogl, Kevin O'Brien, Paul St. Onge

**Affiliations:** United States Army Aeromedical Research Laboratory, Warfighter Performance Group, Fort Novosel, AL, United States

**Keywords:** cognitive workload, support vector machine, operator state monitoring, physiological metrics, multiclass classification, SVM, CWL

## Abstract

**Introduction:**

This study aims to develop and evaluate support vector machines (SVMs) learning models for predicting cognitive workload (CWL) based on physiological data. The objectives include creating robust binary classifiers, expanding these to multiclass models for nuanced CWL prediction, and exploring the benefits of individualized models for enhanced accuracy. Cognitive workload assessment is critical for operator performance and safety in high-demand domains like aviation. Traditional CWL assessment methods rely on subjective reports or isolated metrics, which lack real-time applicability. Machine learning offers a promising solution for integrating physiological data to monitor and predict CWL dynamically. SVMs provide transparent and auditable decision-making pipelines, making them particularly suitable for safety-critical environments.

**Methods:**

Physiological data, including electrocardiogram (ECG) and pupillometry metrics, were collected from three participants performing tasks with varying demand levels in a low-fidelity aviation simulator. Binary and multiclass SVMs were trained to classify task demand and subjective CWL ratings, with models tailored to individual and combined subject datasets. Feature selection approaches evaluated the impact of streamlined input variables on model performance.

**Results:**

Binary SVMs achieved accuracies of 70.5% and 80.4% for task demand and subjective workload predictions, respectively, using all features. Multiclass models demonstrated comparable discrimination (AUC-ROC: 0.75–0.79), providing finer resolution across CWL levels. Individualized models outperformed combined-subject models, showing a 13% average improvement in accuracy. SVMs effectively predict CWL from physiological data, with individualized multiclass models offering superior granularity and accuracy.

**Discussion:**

These findings emphasize the potential of tailored machine learning approaches for real-time workload monitoring in fields that can justify the added time and expense required for personalization. The results support the development of adaptive automation systems in aviation and other high-stakes domains, enabling dynamic interventions to mitigate cognitive overload and enhance operator performance and safety.

## 1 Introduction

Technological advancements have increased functionality and complexity of systems across a variety of domains. However, these advancements often translate to increased burden on the human operator. For example, a system that once required a single operator to *manually control* one system component may now require the operator to *monitor* three automated systems. Monitoring task demands and identifying overload, loss of situational awareness, and/or fatigue can minimize negative impacts of these advanced systems on operator and overall system performance. Operator state monitoring (OSM) research aims to yield a system composed of wearable physiological sensors that feed into machine learning algorithms that ultimately classify an operator's cognitive state. The system would then trigger a countermeasure to prevent performance decline and increased mishap risk. This paper presents the resulting OSM-driven cognitive workload classification algorithms from a novel methodology exploration project that captured physiological data while operators engaged with varying demand levels presented in a low-fidelity aviation-like simulator. Additional information regarding the psychophysical analysis of the performance data from the simulator can be found in Temme et al. (2025) and additional information regarding the desktop simulator utilized in the study can be found in Vogl et al. (2024a).

### 1.1 Cognitive workload assessment

**Figure 1:**
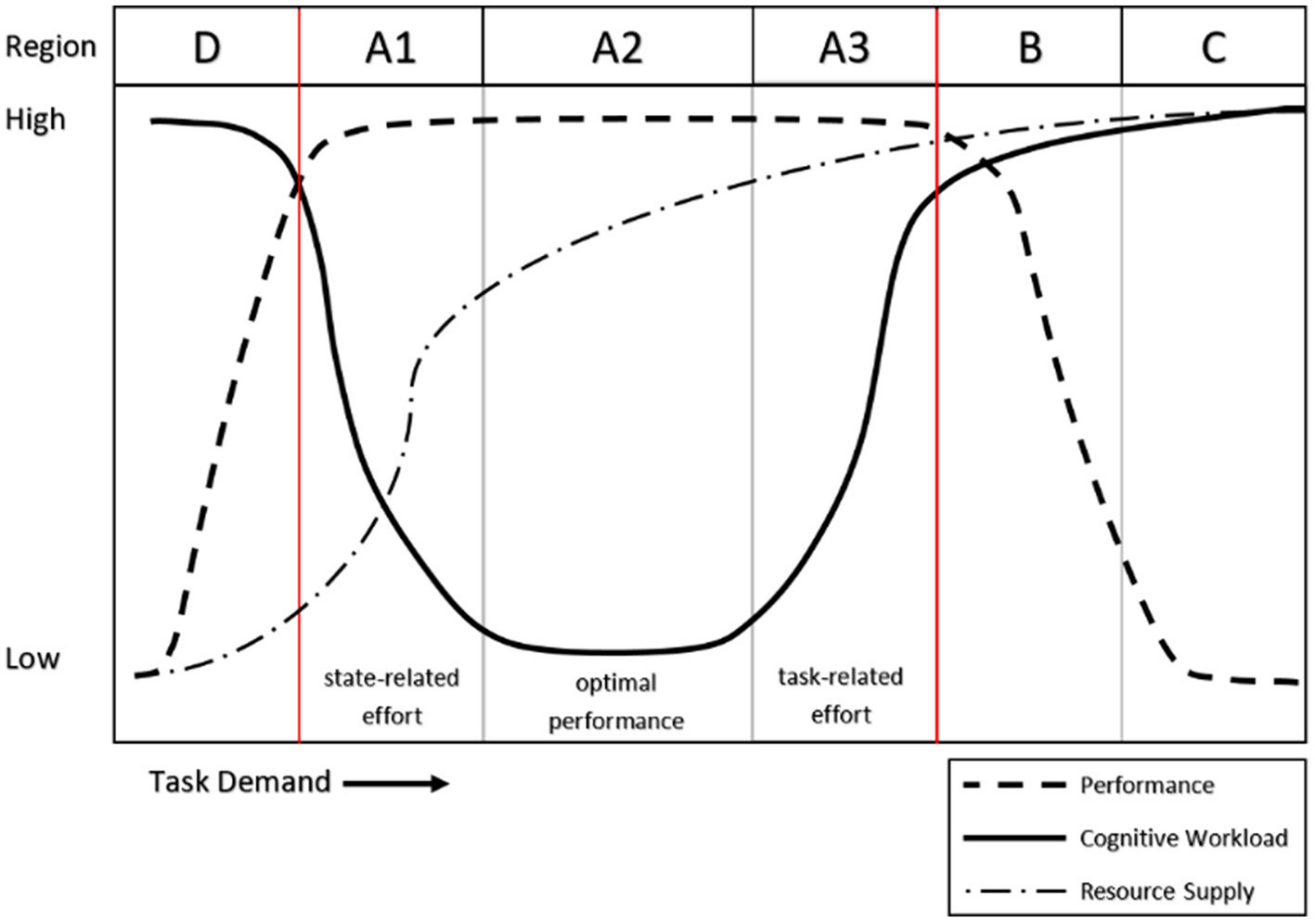
Depiction of the performance-cognitive workload relationship (from Vogl et al., 2022).

Cognitive workload (CWL) is a critical concept in understanding human performance in complex environments, yet a universally accepted definition remains elusive (Cain, 2007). For this work, we adopted the resource-demand framework (Van Acker et al., 2018), which defines CWL as a “subjectively experienced physiological processing state, revealing the interplay between one's limited and multidimensional cognitive resources and the cognitive work demands [these resources are] being exposed to.” This definition encapsulates the core components of CWL: dependence on the interaction between an individual and their environment, roots in resource limitations, and dual manifestation as both a physiological and subjective state. Such a framework builds upon foundational theories of attention and effort (Kahneman, 1973) and resource allocation models, including Multiple Resource Theory (Wickens, 2008), to explore how humans adapt to and are constrained by cognitive demands. The relationship between workload, performance, and resource supply is visualized in the region model depicted in Figure 1.

The operational quantification of CWL has historically relied on three primary measurement domains: task performance, physiological responses, and subjective appraisals. Each domain offers unique advantages and limitations. Performance metrics, for example, provide non-intrusive assessments of task efficiency but often lack the sensitivity to detect subtle changes in cognitive workload, particularly in regions where task performance remains stable (e.g., regions A1, A2, and A3 in Figure 1; Young et al., 2015). Physiological measures, including heart rate variability, pupil diameter, and brain activity metrics like electroencephalography (EEG) and functional near-infrared spectroscopy (fNIRS), allow researchers to observe autonomic nervous system changes in (near) real-time, even during periods where performance metrics may be less sensitive (such as in region A3, Backs, 1995; Aghajani et al., 2017). However, these measures are often susceptible to interference from other physiological states, such as fatigue or anxiety, and may vary in diagnostic precision depending on the metric. Finally, subjective appraisals, such as the widely used multidimensional NASA-TLX scale (Hart and Staveland, 1988) or unidimensional Crew Status Survey (CSS, Ames and George, 1993), provide introspective insights. However, subjective approaches are typically retrospective and may introduce recall biases or cause task interference if completed during task performance.

Given these trade-offs, composite measures combining performance, physiological, and subjective data have emerged as a promising approach to CWL assessment. The concept of association, insensitivity, and dissociation (AID) among these metrics has been explored to better understand inconsistencies and complementarities across measurement domains (Hancock and Matthews, 2019). For example, associations occur when multiple measures indicate converging trends in CWL, while dissociations highlight discrepancies that may provide deeper insights into individual cognitive strategies or measurement limitations. Composite CWL assessments aim to leverage the diagnostic capacity of physiological measures, the contextual relevance of performance metrics, and the introspective accuracy of subjective data (Cain, 2007). Furthermore, the integration of multiple data streams allows researchers to address the multidimensional nature of CWL and better predict transitions toward critical thresholds, such as cognitive overload or performance breakdown (Van Acker et al., 2018; Longo et al., 2022).

### 1.2 Machine learning in OSM

Various machine learning models have been utilized in OSM research, each offering unique advantages and challenges. Deep learning techniques, such as convolutional neural networks (CNNs) and recurrent neural networks (RNNs), have been increasingly popular for processing complex physiological signals. These models excel at identifying subtle patterns in high-dimensional data but often require large datasets, significant computational resources, and can lack interpretability due to their “black-box” nature (Aghajani et al., 2017; Craik et al., 2019). In contrast, traditional machine learning approaches, such as decision trees, random forests, and SVMs, provide more structured and interpretable outputs. Decision trees and random forests offer ease of implementation and intuitive visualization but exhibit high sensitivity to small changes in training data and are typically used for nominal categorization in low-dimensional feature space (Breiman, 2001). SVMs, on the other hand, offer an ideal balance between complexity and performance. They are particularly well-suited for binary and multiclass classification tasks in high-dimensional feature space, where they maximize the margin between decision boundaries, ensuring robust generalization even with limited training data (Cortes and Vapnik, 1995). Unlike deep learning models, SVMs allow for transparent decision-making processes, where the model's outputs can be audited and explained. This capability is crucial in regulatory-heavy industries such as aviation, where understanding why a system made a particular decision is as important as the decision itself.

Specific to OSM research, binary SVM models have been used to classify cognitive workload states based physiological signals, achieving high accuracy with interpretable results (Aricò et al., 2017; Aghajani et al., 2017). Trinary classification tasks, such as distinguishing between low, medium, and high workload levels, have also been effectively implemented using multiclass SVMs (Cervantes et al., 2020). Multiclass SVMs have demonstrated notable promise in OSM, especially for applications requiring nuanced predictions of cognitive states across multiple levels. Unlike binary SVMs, which focus on dichotomous classifications, multiclass SVMs extend this capability to handle more complex classification tasks by employing strategies such as one-vs-rest or one-vs-one. Using a one-vs-rest approach, a separate binary classifier is trained for each class, distinguishing that class from all others, and the class with the highest confidence score among the classifiers is selected as the prediction (Rifkin and Klautau, 2004). The one-vs-one method involves training a binary classifier for every possible pair of classes, and the final prediction is determined by a voting scheme, where the class that wins the most pairwise comparisons is selected (Hsu and Lin, 2002). These approaches enable the modeling of CWL and related states on a continuum, offering granular insights that are critical for real-world applications, such as monitoring fatigue, stress, or workload in high-stakes environments (Cervantes et al., 2020).

One of the primary challenges in implementing multiclass SVMs for OSM lies in their ability to maintain high performance across all classes. Physiological data often exhibits overlapping features between adjacent cognitive states, leading to reduced separability and imbalances in precision and recall across classes (Aricò et al., 2017). Despite these challenges, multiclass SVMs have proven effective in addressing this issue through careful feature selection and preprocessing techniques. For example, hybrid approaches combining SVMs with feature extraction methods, such as principal component analysis (PCA) or wavelet transforms, have enhanced class separability and reduced noise in physiological datasets (Craik et al., 2019; Bashivan et al., 2016).

### 1.3 Study aims

This study aims to build upon this rich body of research by developing and comparing binary and multiclass machine learning models for CWL prediction using physiological data. Specifically, the aims are threefold: (1) to establish robust SVM binary classifiers as a baseline for predicting high versus low CWL states, (2) to expand this framework into multiclass SVM models capable of finer granularity in workload prediction, and (3) to explore the advantages of reduced feature sets and individualized SVM models tailored to specific users. By focusing on key measures, such as task demand and subjective workload ratings, this work seeks to bridge gaps in operational workload assessment and contribute to the development of adaptive systems for complex environments like aviation and healthcare. These models offer the potential to predict workload in (near) real time, thereby enabling preemptive interventions to maintain optimal performance and safety.

For each aim, the models predicted two different values: USAARL MATB demand level and CSS average workload values. The demand level represents the experienced task demand as a function of 10 different demand levels built into the USAARL MATB (see Table 1). This metric served as a tangible ground truth value for what was presented to the subject during the simulation. The CSS average workload value was reported by subjects upon completion of the task, and provided a subjective reflection of what the subject believes they experienced in terms of cognitive workload. The CSS scale ranges from values of 1, easy, to 7, difficult. Accurate predictions of the CSS value would be akin to “reading the subject's mind” by predicting their conscious reflection of the workload they experienced from their unconscious physiology alone.

**Table 1:** Events rates by demand level.

**Task**	**Demand level**									
	**1**	**2**	**3**	**4**	**5**	**6**	**7**	**8**	**9**	**10**
System monitoring	25	30	35	40	45	50	55	60	65	70
Communications	7	8	8	9	9	10	10	11	11	12
RM shutoffs	3	4	5	6	7	8	9	10	11	12
RM failures	6	7	8	9	10	11	12	13	14	15
Tracking	3	4	5	6	7	8	9	10	11	12

Event rates listed for the discrete event tasks (everything but the tracking task) are the number of events per a 5-min period. Tracking indicates the magnitude of the circle motion in the tracking task.

To address the first aim, a standard binary classification model (as often seen in the open literature) was developed to distinguish between high and low levels of workload. To do this, the multiclass nature of the predicted values was compressed into two classes representative of high and low levels of task demand and subjective workload responses. For the binary demand level classifier, high and low demand conditions were created by parsing the data into two categories with demand levels 1–3 representing low demand and demand levels 8–10 representing high demand. This level of separation is consistent with the JND values observed in the USAARL MATB Temme et al. (2025). The CSS values were binarized by separating responses <5 representing low workload and 5 or greater representing high workload. This separation boundary was chosen as it identifies when subjects were approaching performance decrements as indicated by the description accompanying the CSS rating of 5.

The second aim focused on expanding the modeling effort to a multiclass classifier using the full dataset. This approach explored the feasibility of predicting CWL at a finer resolution. Such a model offers deeper insights into the dynamics of workload transitions, enabling the detection of subtle shifts in cognitive demand that signal an approach to the “redline”—the point at which workload exceeds manageable levels. By leveraging physiological data alone, this effort not only assessed the predictive power of such models but also examines their practical utility in scenarios where subjective self-reports or external workload indicators may not be available. This finer resolution of prediction could lead to the development of proactive workload management systems, capable of dynamically adapting to individual needs in real time, improving safety, efficiency, and performance across various domains, such as aviation, healthcare, and other high-stakes decision-making environments.

The third aim focused on developing individualized models to enhance predictive accuracy, representing a critical step forward in tailoring CWL prediction systems. These models were personalized by accounting for individual differences among subjects and incorporating feature importance to refine the selection of relevant physiological metrics. Individualizing models addressed the inherent variability in human responses to CWL, which can be influenced by factors such as cognitive capacity, stress tolerance, and baseline physiological states. Such advancements have significant implications for real-world applications, enabling systems to provide more reliable and user-specific insights, particularly in high-stakes environments where CWL management is critical. This effort also contributes to the broader understanding of how physiological data reflects workload at an individual level, paving the way for innovations in adaptive training, operational safety, and performance optimization.

## 2 Method

A detailed description of the methodology and experimental setup employed to collect the data utilized in the classification model can be found in Temme et al. (2025). A brief summary of the methodology as it pertains to the development of the CWL classification models is provided here.

### 2.1 Subjects

A total of 3 subjects, 1 female and 2 males, participated in the study. The subjects' ages ranged from 24 to 47. The small number of subjects was balanced by the large number of simulation trials that each subject completed. Subjects engaged in enough trials to result in 6.8 to 10.8 h of data collection per subject. Subjects included research team members, as traditionally is the case in the psychophysical assessment context the data was collected under (see Temme et al., 2025). Subjects were non-military civilians with no aviation experience (as no aviation experience was required to perform the task).

U.S. Army Medical Research and Development Command Institutional Review Board reviewed and approved this protocol (USAARL number 2020-013) on 21 Jan 2021 under number M-10881. Before any testing, each volunteer went through the informed consent process individually in a private setting with a properly credentialled study team member.

### 2.2 Materials

#### 2.2.1 USAARL MATB

The low-fidelity aviation-like simulator employed for this study was the United States Army Aeromedical Research Laboratory Multi-Attribute Task Battery (USAARL MATB). The USAARL MATB, created by Vogl et al. (2024b), is a custom designed version of the MATB originally developed by NASA (Comstock and Arnegard, 1992). The USAARL MATB was designed to both replicate the original tasks utilized in the original MATB while also enabling more efficient parameter generation, data analysis, and integration of customized features. The USAARL MATB features four subtasks: system monitoring, communications, tracking, and resource management. Subjects interact with the tasks using a joystick (system monitoring and tracking) and a mouse (communications and resource management). While the USAARL MATB has the capability for subtasks to be automated, no automation systems were allowed to be employed during this study. Custom modifications for loading the experiment specific parameter files were made for the execution of the study, but these modifications did not change the nature of the original NASA MATB subtasks. For a review of additional details on the functionality of the USAARL MATB, see Vogl et al. (2024b). Each trial of the USAARL MATB lasted 5-min. Performance data, task markers, and the task demand level was automatically streamed through a network outlet managed by Lab Streaming Layer (LSL; Kothe et al., 2024) software for recording and synchronization with physiological metrics.

While the USAARL MATB does allow for custom modifications of the task demand presented across all the tasks, only the 10 default demand levels were utilized in this study (Table 1). Demand level is defined by event rates for the discrete event tasks throughout the simulation. Additionally, the continuous rate at which the target circle for the tracking task randomly drifts increases with each demand level step. Demand level was recorded for each simulation to serve as a target variable for model prediction.

#### 2.2.2 Pupillometry

Pupil diameter was measured using the Pupil-Labs Core Binocular (Pupil-Labs, Berlin, Germany) head-mounted eye tracking system; a video-based, infrared (860 nanometer) system that utilizes two head-mounted eye cameras suspended just below the left and right eyes. The pupil was identified and measured using a custom algorithm developed by Pupil Labs (Kassner et al., 2014). The subject completed a five-point calibration process prior to each session to allow the system to calibrate a 3-dimensional (3D) model of the eye relative to the subject's eye measurements. Due to the eye camera being fitted to the subject's head (akin to wearing glasses), the distance between the subject's eye and the eye camera remained constant. This prevented measurement error of pupil size caused by movement closer to or further from the camera. Any change in this distance (such as the subject accidentally bumping the camera during task engagement) was compensated for by the 3D model used in the algorithm and was flagged appropriately. The eye cameras recorded the subject's pupil size using 400 × 400-pixel resolution eye images to derive a pupil diameter measurement in pixels (and convert it into millimeters [mm]) at a sample rate of 120 Hz.

#### 2.2.3 Electrocardiogram (ECG)

ECG was recorded continuously, at a sampling rate of 512 Hz, using the Shimmer 3 composed of four leads to electrodes adhered to the subject's chest, specifically, on the clavicles and one electrode between the sixth and seventh intercostal space on each the left and right sides of the torso. The Shimmer 3 used a Bluetooth connection from a small, battery powered, subject-worn transmitter to send data to the computer.

#### 2.2.4 Crew Status Survey (CSS)

The CSS is a CWL assessment scale, originally validated and verified using trained pilots and aircrew members (Ames and George, 1993). Operators are asked to assess their perceived average CWL level using a response on a seven-point scale (1 = low workload; 7= high workload; Table 2). The CSS was administered at the completion of each 5-min trial. The average subjective CWL value was of primary interest for analysis and served as a target variable for the resulting SVM classifiers.

**Table 2:** Anchor number and description used in the CSS.

**Level**	**Description**
1	Nothing to do; no system demands
2	Light activity; minimal demands
3	Moderate activity; easily managed considerable spare time
4	Busy; challenging but manageable; adequate time available
5	Very busy; demanding to manage; barely enough time
6	Extremely busy; very difficult; non-essential tasks postponed
7	Overloaded; system unmanageable; essential tasks undone; unsafe

### 2.3 Procedure

Subjects were research team members who were instrumented with the eye tracking and ECG physiological sensors for each session. Each subject would schedule time for a data collection session, with each session typically lasting between 70 and 90 min (amounting to roughly 14 to 18 trials per session). Subjects' input their subject ID and the number of trials they wanted to complete, pressed “start”, and then began a series of simulation trials presented in sequential order. Trial duration was 5 min and demand level was randomly assigned. The task demand was modulated across successive trials. During each trial, data was captured and synchronized using LSL, which compiles and saves the data (along with timing information for synchronization) in one master file. Each trial's data included performance data from the USAARL MATB, physiological recordings from the eye tacking and ECG systems, and a subjective appraisal of workload using the CSS following completion of each trial.

### 2.4 Physiological feature extraction

In preparation for model development, a custom data processing and feature extraction pipeline was developed using the Python language. The LSL output containing event markers and physiologic recordings was an XDF file. Using the pyxdf (v.1.16.3; Boulay et al., 2020) library, each XDF file was converted into a set of dictionaries containing each stream of data that Lab Recorder (an LSL software) stored. Opening these files required setting the flags for “synchronize_clocks,” “handle_clock_resets,” and “dejitter_timestamps” to true when opening the physiological data to apply the appropriate synchronization across the different physiological devices.

Custom classes were written to parse the USAARL MATB, ECG, CSS, and pupillometry recordings. Physiological recordings were segmented using a sample window of approximately 12.3 s (based on trial segmentation employed for other tasks performed within the laboratory this work was conducted). Although the USAARL MATB does not have regular stimulus intervals, the start-time of a trial was pulled from the recording and a matching temporal segmentation was performed using an assumed constant 5-stimulus window of 2.46 second intervals. This allowed the physiological processing pipeline to be utilized across multiple task analyses. The parsers also aggregated any needed information from matching text files, extracted and parsed the subject IDs, task settings, CSS ratings, and stored filenames for linking between sources of summary data.

The ECG recordings were extracted from the XDF file and reformatted into a data frame using the Pandas (v1.1.3; McKinney, 2010) library. Because the ECG recordings include waveforms from multiple leads, preliminary visualizations of the raw ECG traces were used to visually identify a channel which presented minimal high frequency noise, and the trace from that channel was consistently used throughout the analysis. The R-wave peaks were identified in the ECG trace using the Neurokit2 library (v0.2.0; Makowski et al., 2021). The time difference between successive peaks was used to calculate inter-beat intervals (IBIs) which were fed into the hrvanalysis (v1.0.3; Champseix et al., 2021) library to generate a set of commonly used heart rate variability (HRV) metrics. Leveraging this library, provided time domain features (mean NNI, SDNN, SDSD, NN50, pNN50, NN20, pNN20, RMSSD, median NN, range of NN, CVSD, CV NNI, mean HR, max HR, min HR, and standard deviation HR), geometrical domain features (triangular index, and TINN), frequency domain features (LF, HR, VLF, LH/HF ratio, LF nu, HF nu, total power), and non-linear domain features (CSI, CVI, modified CSI, SD1, SD2, SD1/SD2 ratio, sample entropy). These features were output as an exploratory examination of which ECG features may be important in cognitive workload prediction. These features were down selected to relevant cognitive workload metrics known to correlate to changes in ECG signals (e.g., heart rate, heart rate variability, ECG frequency information, etc.; Vogl et al., 2020).

Tabular pupil diameter values were extracted from the pupillometry channel of the XDF recordings and converted to Pandas data frames. A custom filter was developed to drop values representing blink artifacts, loss of tracking, or other sources of error. Differences in successive diameters were first divided by differences in successive timestamps to calculate a velocity metric. This process was repeated with successive velocities to calculate an acceleration metric. Two masking variables were created, the first identified frames where the measured pupil diameter was below 1.0 mm and the second labeled frames where the absolute value of the calculated acceleration exceed 10,000 millimeters per squared second (mm/s^2^). For each frame in a recording, the Boolean OR of these two masking variables was used to generate a variable representing a pupillometry failure, and adjacent frames were additionally marked. Any frame marked as a pupillometry failure or adjacent to a pupillometry failure was then marked and dropped. Leveraging the original indices from the Pandas data frames, lengths of sets of frames with sequential indices were calculated and any set with fewer than 50 sequential frames was dropped. After this filtering, right and left eye pupil diameter metrics (mean, median, minimum, maximum, standard deviation, and kurtosis) were calculated.

For each session, the task metrics described above were aggregated into a CSV file. Within a recording, for each moving window, the HRV and pupillometry metrics were calculated for that time period and stored as successive rows in spreadsheets. In both these intermediate analysis outputs, the corresponding XDF filename was incorporated as a column, which permitted joint operations to be performed at the model construction stage.

### 2.5 CWL model development

The goal of model development for this project was the *prediction* of observed: (1) objective USAARL MATB task demand level and (2) subjective CSS ratings directly *from physiological sensor data* (ECG and pupillometry). Given the long-term operational goal of real-time estimation of cognitive workload in military aviators, there were two additional constraints. First, any models developed need to leverage techniques that are non-proprietary to be modular open system approach (MOSA) compliant. Second, any models must have fully auditable behavior, as this is a regulatory requirement for any major aviation sub-system. Given these constraints, the models developed for this effort were SVMs.

SVMs were built, trained, tested, and validated using the scikit-learn (v.1.1.1; Pedregosa et al., 2011) library. A one-vs-rest classifier method was used with a maximum of 2,000 training iterations. Hyperparameters for regularization, kernel type, and kernel coefficient were iteratively tested using a grid search function, which tests all permutations of specified hyperparameter options. Initial exploratory testing showed better performance when using the radial basis function (RBF) kernel than with a linear, second-order polynomial, or third-order polynomial kernel under all conditions, so only the RBF kernel was used for final model construction for both tasks. For final MATB modeling, values for the “C” hyperparameter (which is the inverse of regularization strength) were 1 and 5 through 50 in increments of 5 while values for “gamma” (kernel coefficient) were 0.1 to 1.0 in steps of 0.1. The range of hyper parameters were chosen to balance the thoroughness of the grid-search approach against the realistic limits of available computational resources and analysis time. At the extreme values of our chosen hyperparameter range, it was common to either hit the 2,000 training iteration maximum without converging to stable support vectors or to quickly converge with poor classifier performance. More optimizations can be achieved in future iterations of this work by using more sophisticated search algorithms. For each grid search, the best performing hyperparameters are included in tabular form for comparison or replication (Table 3).

**Table 3:** Grid search hyperparameters for best fitting models.

**Model**	**Feature set**	**Subject(s)**	**C**	**Gamma**
Binary CSS	All	1, 2, and 3	5	0.6
	Top 10	1, 2, and 3	5	1
	Classic CWL	1, 2, and 3	1	1
Binary DL	All	1, 2, and 3	5	0.7
	Top 10	1, 2, and 3	10	1
	Classic CWL	1, 2, and 3	5	0.9
Multiclass CSS	All	1	5	0.2
	All	2	5	0.1
	All	3	5	0.1
	All	1, 2, and 3	1	0.5
	Top 10	1	5	0.6
	Top 10	2	20	0.7
	Top 10	3	10	0.7
	Top 10	1, 2, and 3	1	0.7
	Classic CWL	1	10	0.5
	Classic CWL	2	25	0.5
	Classic CWL	3	10	0.5
	Classic CWL	1, 2, and 3	1	0.5
Multiclass demand level	All	1	5	0.2
	All	2	20	0.1
	All	3	10	0.1
	All	1, 2, and 3	5	0.5
	Top 10	1	10	0.7
	Top 10	2	20	0.7
	Top 10	3	20	0.7
	Top 10	1, 2, and 3	10	0.7
	Classic CWL	1	20	0.5
	Classic CWL	2	50	0.5
	Classic CWL	3	25	0.5
	Classic CWL	1, 2, and 3	10	0.5

Both binary and multiclass SVMs were constructed for each individual subject as well as an aggregate across subjects. The SVM utilized a balanced weighting approach to account for the potential variance in the number of trials performed across subjects. The USAARL MATB demand level and the CSS average workload score were binarized and used as a label for each of the individual members of the one-vs-rest classifiers. The pupillometry and heart rate variability metrics for each of approximately 12.3 s moving windows were used as features for the model training after dropping TINN (which was returned as missing data by the hrvanalysis library because the TINN calculation requires longer durations of IBIs). Additionally, rows with missing data were dropped, as SVMs cannot be trained without imputing missing values, and imputation of variability metrics is methodologically unsound. An 80:20 train/validate split was performed prior to the grid search and each model in the grid search was trained using a 5-fold cross-validation. After the grid search, the highest accuracy model was used to conduct predictions by taking the label of the individual classifier in the one-vs-rest set with the highest calculate probability of a positive observation. This ensured a single predicted MATB demand level, and the CSS average rating were returned for each of the moving windows in the testing set. The highest accuracy model, as defined by the hyperparameters presented in Table 3, was validated utilizing a 5-fold cross validation approach to obtain a distribution of model accuracy for further assessment.

From each model, feature importance was derived utilizing a model-agnostic approach that evaluates the importance by quantifying how much the model's performance decreases when a feature's values are randomly shuffled. Multiple permutations of the model were performed with modified versions (i.e., randomly shuffled) of a feature to essentially break its association with the target variable. Model performance with the modified feature was then compared against baseline to determine how much of a performance drop occurred due to the modification of the feature. Larger drops in performance indicate higher levels of importance the original feature had within the original model. This process was repeated multiple times across all features to derive a rank ordering of feature importance for each model. The resulting rank order provided means to conduct the same approach to building a second model that utilized only the top 10 most important features for continued refinement and understanding of the classification problem.

The predicted USAARL MATB demand level and CSS average were matched with the actual USAARL MATB demand level and reported CSS workload ratings to generate a confusion matrix for the construction of accuracy, precision, recall (also termed as sensitivity or the true positive rate), and Matthews Correlation Coefficient (MCC) values to assess final model performance. The MCC is a single metric that evaluates the quality of classifications by considering true and false positives and negatives, with values ranging from −1 (perfect disagreement) to 1 (perfect agreement), and 0 indicating no better than random prediction. Additionally, receiver operating characteristic (ROC) curves and the area under these curves (AUC-ROC) were generated and plotted for each model as this is a standard representation of model skill across machine learning techniques. The area under the AUC-ROC represents the average ability of the classifier to distinguish between each pair of classes; here it is calculated as a macro-average using a one-vs-rest scheme. For each individual classifier, precision [true positives / (true positives + false positives)], recall [true positives / (true positives + false negatives)], AUC-ROC, and MCC values are derived for both demand level and CSS rating predictions.

Although there are 10 possible demand levels for the MATB and 7 possible ratings for workload, not all values were experienced and/or reported by all subjects. When calculating fitness metrics for each model, rows and columns of zeros were inserted where appropriate to ensure identical dimensions before merging. Additionally, to make comparisons across sample sizes more intuitive, the confusion matrices were converted from counts to proportions before the fitness metrics were calculated (Note: Neither of these procedures impacts the calculated model fitness metrics.).

The same approach was utilized to provide classification results with more simplified models that were calibrated by the feature importance results of the feature rich SVM previously utilized. The top 10 rank-ordered features were selected for each subject and the combined subject models. This means, the resulting model was tailored specifically to each subject (and combination of subjects) based on their unique physiological importance responses. These top features were included in the feature set to train a new series of SVMs to predict the same demand level and CSS values. As a final complexity reduction, a third feature set was developed based on common metrics utilized in the field of cognitive workload assessment. The included features in the third feature set were: right and left pupil diameter means, mean heart rate, two heart rate variability metrics (root mean square of successive differences [RMSSD] and the ratio between low and high frequency cardiac data [LF-HF ratio]). Three feature sets (All Features, Top 10 Features, and Classic CWL Metric Features) were used across models to predict demand level and CSS values.

## 3 Results

### 3.1 Binary classification model performance

The resulting confusion matrices for the binary demand level and CSS prediction models are presented in Table 4. The confusion matrices provide the proportion of observations from the reserved testing data that correspond to each combination of truth (rows; i.e., the demand level experienced in the USAARL MATB and the participant's reported average workload rating on the CSS) and classifier prediction (columns). True positives occur when the truth and prediction are matched, and these occur diagonally across the matrix. The values listed in Table 4 are proportions, rather than counts, the sum of the matching truth/prediction values along the diagonal is the exact match accuracy of the model.

**Table 4:** Confusion matrices for binary classification models.

**Model**	**True value**	**All feature Model prediction**		**CWL metrics Model prediction**	
		**Low DL**	**High DL**	**Low DL**	**High DL**
Demand level	True Low DL	**0.4658**	0.0716	**0.4425**	0.0995
	True High DL	0.2186	**0.2394**	0.2971	**0.1609**
**Model**	**True value**	**All feature Model prediction**		**CWL metrics Model prediction**	
		**Low CSS**	**High CSS**	**Low CSS**	**High CSS**
CSS	True Low CSS	**0.6764**	0.0343	**0.6637**	0.0470
	True High CSS	0.1615	**0.1278**	0.1624	**0.1269**

Bold values highlight correct predictions.

The two SVM models for demand level and CSS predictions demonstrated fair (AUC-ROC: 0.70–0.80) to good (AUC-ROC: 0.80–0.90) discrimination between binary classes, highlighting the models' ability to distinguish between categories effectively. The resulting test scores of the binary classification models are summarized in Table 5. For demand level prediction, the model achieved an accuracy of 0.7052 (Recall: 0.6911; Precision: 0.7196; AUC-ROC: 0.77; MCC: 0.4097) when using all available features to discriminate the data, demonstrating strong predictive performance. However, accuracy dropped to 0.6034 [Recall: 0.5839; Precision: 0.6081; AUC-ROC: 0.64, indicating poor discrimination (0.50–0.70); MCC: 0.1905] when relying solely on the classic CWL metric feature set. Gross subjective CSS class prediction achieved higher accuracy overall, with the model reaching 0.8042 (Recall: 0.6967; Precision: 0.7977; AUC-ROC: 0.84; MCC: 0.4840) using all features and maintaining strong performance at 0.7906 (Recall: 0.7666; Precision: 0.6863; AUC-ROC: 0.75; MCC: 0.4456) when using the reduced classic CWL metric feature set.

**Table 5:** Performance metrics for USAARL MATB binary classifiers across all subjects.

**Model features**	**Metric**	**Demand level (DL1-3 vs. DL8-10)**	**CSS CSS < 5 vs. CSS≥5**
All	Accuracy (Exact)	0.7052	0.8042
	Recall	0.6911	0.6967
	Precision	0.7196	0.7977
	AUC-ROC	0.77	0.84
	MCC	0.4097	0.4840
Top 10	Accuracy (Exact)	0.6458	0.8111
	Recall	0.6329	0.7489
	Precision	0.6481	0.7747
	AUC-ROC	0.65	0.83
	MCC	0.4641	0.5230
Classic CWL metrics	Accuracy (Exact)	0.6034	0.7906
	Recall	0.5839	0.7666
	Precision	0.6081	0.6863
	AUC-ROC	0.64	0.75
	MCC	0.1905	0.4456

Recall and precision are calculated as macroaverage values.

**Figure 2:**
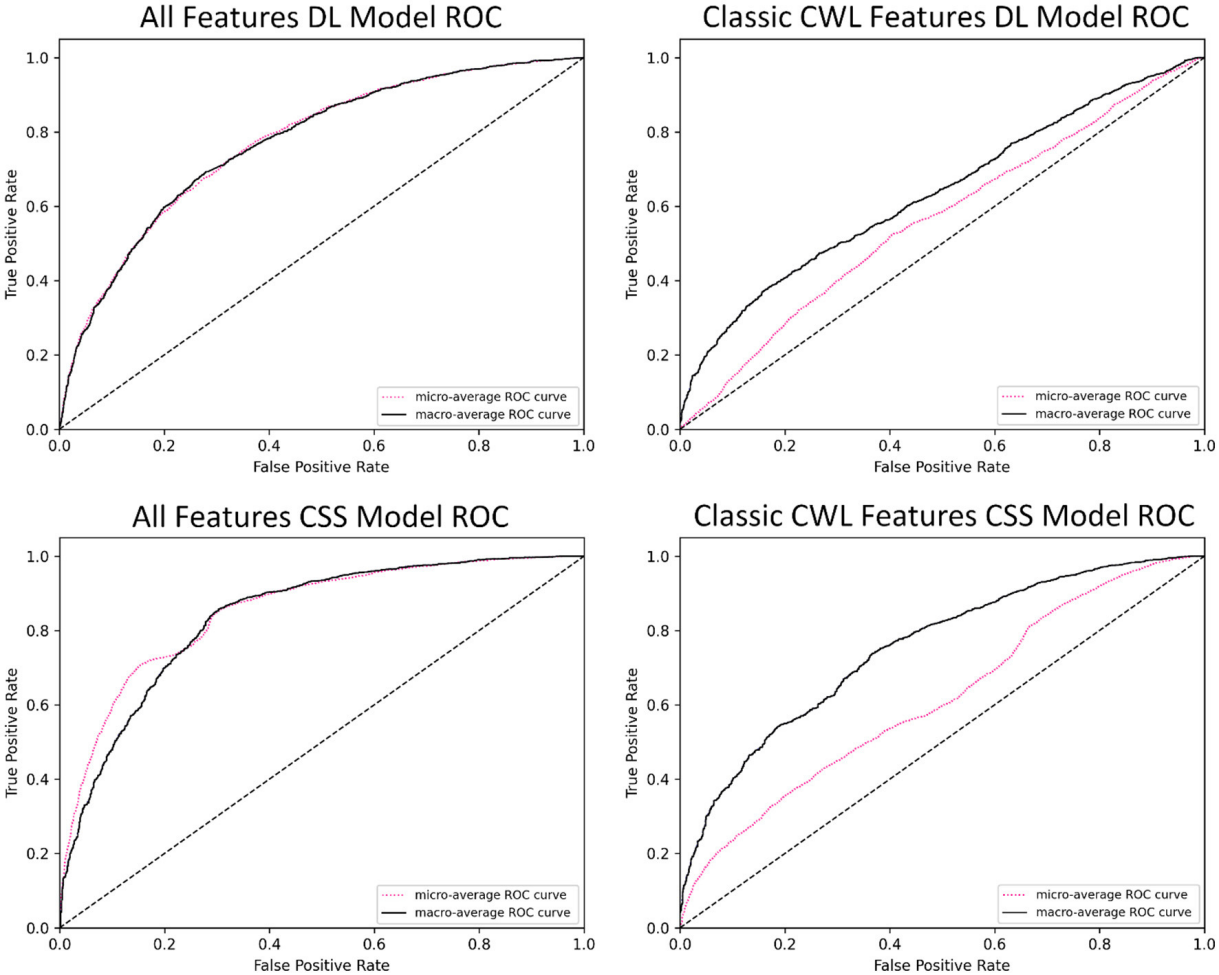
Exemplar ROC visualizations of the binary CSS classifiers for the complete and CWL metric feature sets.

ROC visualizations are included (Figure 2) for each classifier, including micro- and macro-average curves. The ROC curve illustrates the trade-off between the true positive rate (sensitivity) and the false positive rate (1-specificity) across varying classification thresholds. These visualizations are typically interpreted relative to a diagonal line (which is included) that represents a model with skillless performance (i.e., random chance predictions). A perfectly predictive model would be represented by a vertical line rising from the origin and a horizontal line starting at the point (0,1) and continuing to the point (1,1), indicating perfect discrimination between classes. The area under the curve (AUC), included in the legend, quantifies the overall performance of the model, with a value of 1.0 indicating perfect prediction performance and 0.5 indicating completely random prediction performance. The ROC visualization intuitively highlights the performance between the model's tasks and allowed feature sets. The AUC-ROC for the demand level classifier drops from 0.77 to 0.64 between the feature sets. In fact, the ROC presented for the classic CWL metric feature set in Figure 2 demonstrates how poorer performance leads to the curve falling toward the diagonal. On the other hand, the AUC-ROC for the CSS classifier 0.84 to 0.75 between the feature sets, generally maintaining predictive accuracy.

**Figure 3:**
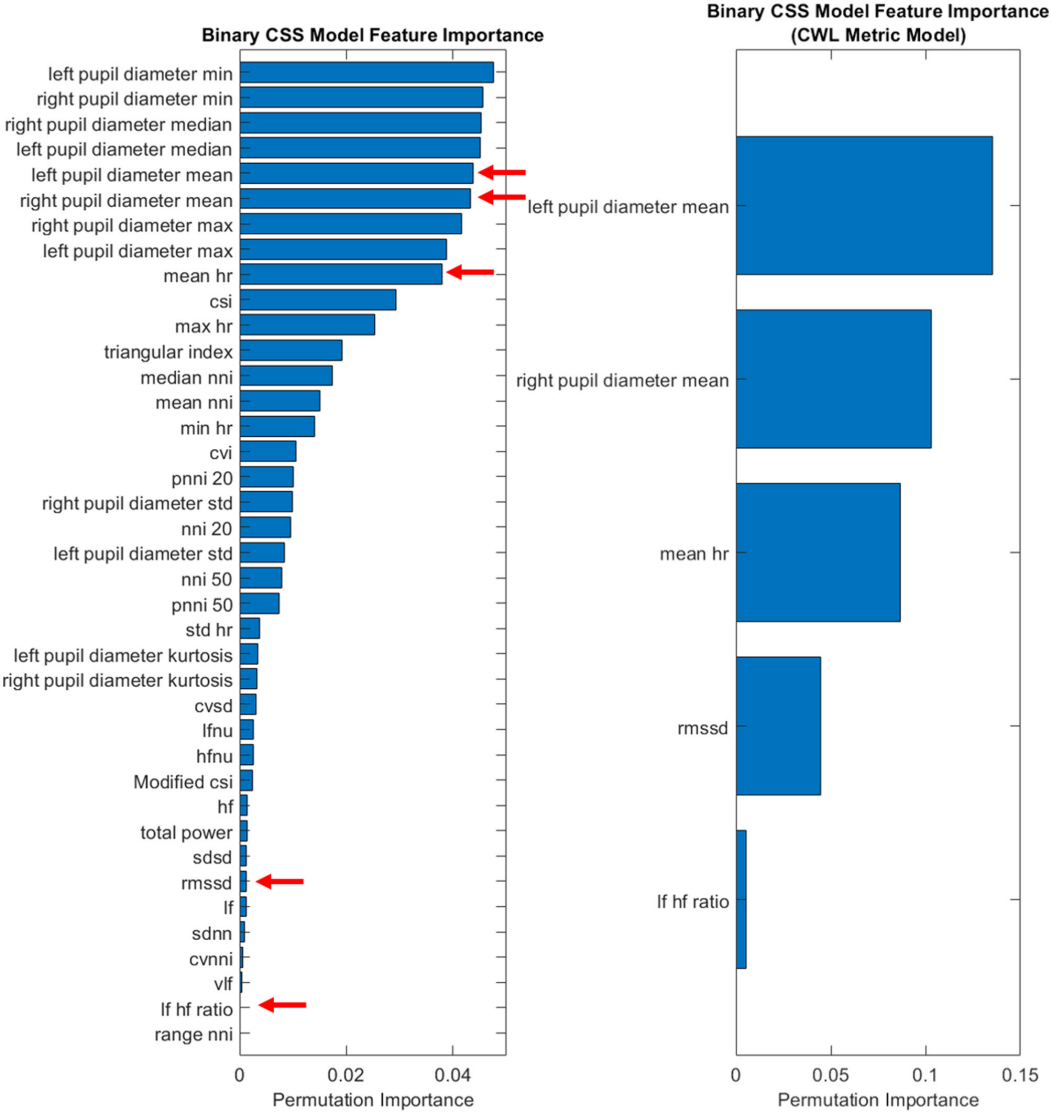
Feature importance for binary CSS model.

Given the limitations of the demand level prediction model when reduced to the smaller feature set, only the CSS classifier was examined in more depth. Of particular interest is the performance of the CSS prediction model in identifying when subjects would be likely to report that the demand they experienced was at a 5 or higher on the CSS (a threshold indicating high cognitive workload). While a statistically significant drop in performance, *t*_(4)_ = 3.4, *p* = 0.028, was observed between the complete feature set (*M* = 0.8217, *SD* = 0.0062) and the classic CWL metric feature set (*M* = 0.7960, *SD* = 0.0153), the minor deficit should be examined with an eye toward the benefits of reduced complexity of the classifier. To further explore the CSS model's feature contributions, an importance permutation was conducted. The resulting feature importance ranking is depicted in Figure 3. The shift from the entire feature set of 39 physiological features was reduced to a total of 5 classic cognitive workload metrics, representing a large reduction in model complexity while yielding only minor reductions in performance. Of particular note, the feature importance of the heart rate variability metrics appears to be quite small relative to mean heart rate and the pupil diameter means. This implies that the HRV metrics that were expected to be important features *a priori* may be less important to cognitive workload prediction in the evaluated context.

### 3.2 Multiclass classification model performance

The multiclass classifier was tested to determine the accuracy with which fine resolution predictions could be made and utilized. Methodological deviations from the binary approach are highlighted here, but the same structure of results and visualizations are presented. In the multiclass prediction, the truth space expands from two possible states to 10 states for the demand level classifier and 5 states for the CSS classifier. As such, the random chance of the classifier to correctly predict the true state drops from the 0.50 chance at the binary level to 0.10 for the demand level predictions and 0.2 for the CSS value predictions. Accuracy values were assessed against these adjusted levels of random chance.

The included confusion matrices (Tables 6, 7) show the proportion of observations from the reserved testing data that correspond to each combination of truth and classifier prediction. Missing values, represented with “not applicable (N/A),” occur when there were no ratings of the corresponding value given by the subjects. In this case, subjects did not utilize the full CSS scale when making their subjective workload judgements, omitting the use of ratings 1 and 7 completely.

**Table 6:** Confusion matrix–USAARL MATB for predicting USAARL MATB demand level.

**True value**	**Prediction**									
	**1**	**2**	**3**	**4**	**5**	**6**	**7**	**8**	**9**	**10**
True 1	**0.1222**	0.0109	0.0081	0.0123	0.0046	0.0075	0.0075	0.0121	0.0075	0.0084
True 2	0.0293	**0.0418**	0.0029	0.0023	0.0013	0.0033	0.0015	0.0042	0.0048	0.0012
True 3	0.0178	0.0046	**0.0211**	0.0042	0.0012	0.0023	0.0044	0.0058	0.0019	0.0017
True 4	0.0255	0.0050	0.0019	**0.0433**	0.0038	0.0046	0.0050	0.0082	0.0067	0.0023
True 5	0.0184	0.0017	0.0017	0.0021	**0.0270**	0.0017	0.0004	0.0042	0.0019	0.0023
True 6	0.0253	0.0046	0.0017	0.0033	0.0021	**0.0301**	0.0023	0.0065	0.0040	0.0013
True 7	0.0230	0.0050	0.0029	0.0029	0.0008	0.0031	**0.0362**	0.0038	0.0038	0.0035
True 8	0.0357	0.0054	0.0033	0.0071	0.0036	0.0044	0.0035	**0.0692**	0.0058	0.0036
True 9	0.0249	0.0042	0.0017	0.0067	0.0013	0.0023	0.0056	0.0073	**0.0433**	0.0013
True 10	0.0180	0.0013	0.0025	0.0040	0.0019	0.0008	0.0017	0.0021	0.0008	**0.0336**

Bold values highlight correct predictions.

**Table 7:** Confusion matrix–USAARL MATB for predicting CSS average workload score.

**True value**	**Prediction**						
	**1**	**2**	**3**	**4**	**5**	**6**	**7**
True 1	**N/A**	N/A	N/A	N/A	N/A	N/A	N/A
True 2	N/A	**0.0397**	0.0245	0.0650	0.0186	0.0033	N/A
True 3	N/A	0.0084	**0.0924**	0.0894	0.0332	0.0058	N/A
True 4	N/A	0.0119	0.0301	**0.1889**	0.0395	0.0054	N/A
True 5	N/A	0.0015	0.0184	0.0807	**0.1548**	0.0063	N/A
True 6	N/A	0.0019	0.0052	0.0280	0.0144	**0.0326**	N/A
True 7	N/A	N/A	N/A	N/A	N/A	N/A	**N/A**

Bold values highlight correct predictions.

The overall accuracy of the combined subject model for predicting USAARL MATB demand level was 0.4680 (Recall: 0.4458; Precision: 0.5052; AUC-ROC: 0.79; MCC: 0.3924); while the overall accuracy of the model for predicting CSS responses was 0.5061 (Recall: 0.4681; Precision: 0.5507; AUC-ROC: 0.75; MCC: 0.3553). The resulting accuracy metrics can be found in Tables 8, 9. The reported recall and precision are the macroaverages of the class-wise precision and recall, respectively. There is no included microaverage precision or recall because those metrics are identical to accuracy in a multiclass classification where all observations are predicted as a single class.

**Table 8:** Performance metrics for USAARL MATB classifiers showing accuracy for predicting USAARL MATB demand level when using all features.

**Model features**	**Metric**	**Subject ID**			
		**1**	**2**	**3**	**1**+**2**+**3**
All	Accuracy (Exact)	0.5627	0.5621	0.5517	0.4680
	Accuracy (±1)	0.6355	0.6610	0.6498	0.5595
	Recall	0.5350	0.4610	0.5065	0.4458
	Precision	0.5945	0.5349	0.5391	0.5052
	AUC-ROC	0.89	0.86	0.88	0.79
	MCC	0.4970	0.4658	0.4934	0.3924
Top 10	Accuracy (Exact)	0.6218	0.6271	0.7048	0.5123
	Accuracy (±1)	0.6897	0.7232	0.7799	0.5894
	Recall	0.5992	0.5519	0.6936	0.5199
	Precision	0.6307	0.5779	0.7049	0.5329
	AUC-ROC	0.91	0.91	0.95	0.87
	MCC	0.5667	0.5495	0.6674	0.4499
Classic CWL metrics	Accuracy (Exact)	0.5627	0.5749	0.5344	0.3368
	Accuracy (±1)	0.6355	0.6808	0.6392	0.4647
	Recall	0.5213	0.4852	0.5239	0.3458
	Precision	0.6150	0.5596	0.5478	0.3645
	AUC-ROC	0.90	0.91	0.89	0.77
	MCC	0.4973	0.4832	0.4750	0.2546

Recall and precision are calculated as macroaverage values.

**Table 9:** Performance metrics for USAARL MATB classifiers showing accuracy for predicting a CSS rating when using all features.

**Model features**	**Metric**	**Subject ID**			
		**1**	**2**	**3**	**1**+**2**+**3**
All	Accuracy (Exact)	0.6543	0.6455	0.6086	0.5061
	Accuracy (±1)	0.8242	0.9053	0.8297	0.8067
	Recall	0.6128	0.5658	0.6040	0.4681
	Precision	0.6636	0.6678	0.6062	0.5507
	AUC-ROC	0.86	0.84	0.85	0.75
	MCC	0.5361	0.4628	0.4965	0.3553
Top 10	Accuracy (Exact)	0.6880	0.6525	0.7435	0.4141
	Accuracy (±1)	0.8477	0.9082	0.8732	0.7532
	Recall	0.6437	0.5759	0.7397	0.3732
	Precision	0.6744	0.6525	0.7326	0.4414
	AUC-ROC	0.89	0.87	0.93	0.87
	MCC	0.5819	0.4758	0.6712	0.2323
Classic CWL metrics	Accuracy (Exact)	0.6103	0.6412	0.6153	0.3857
	Accuracy (±1)	0.8113	0.8715	0.8344	0.7200
	Recall	0.5682	0.5483	0.6153	0.3537
	Precision	0.6152	0.6605	0.6195	0.4325
	AUC-ROC	0.85	0.86	0.86	0.67
	MCC	0.4789	0.4556	0.5044	0.2023

Recall and precision are calculated as macroaverage values.

#### 3.2.1 Individualized models

**Figure 4:**
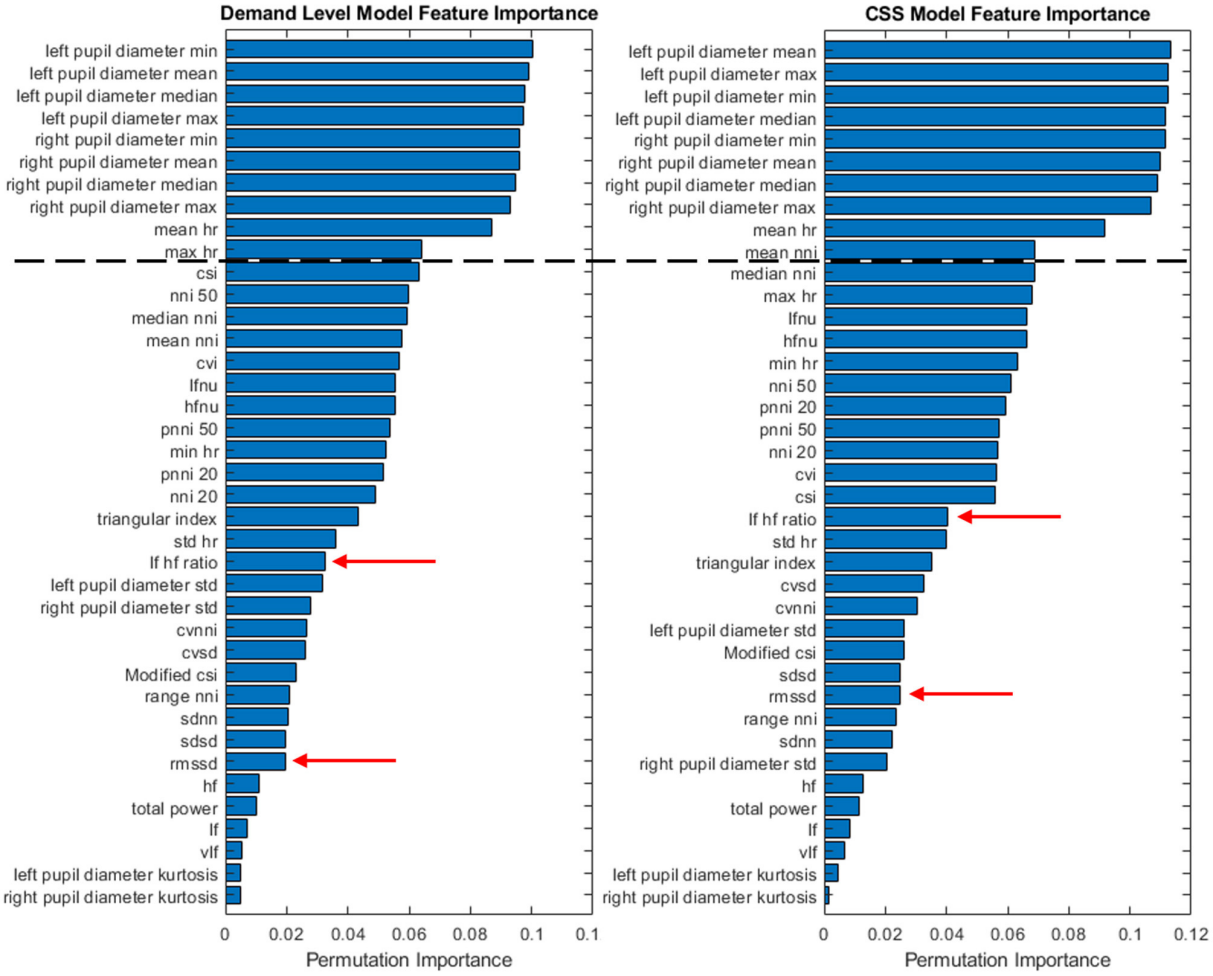
Feature importance for each classifier type across all subjects (S1 + S2 +S3).

The confusion matrix values presented in Tables 6, 7 represent the accuracy based on a model derived from all three subjects. In an effort to explore between subject differences, individualized models were developed utilizing the same SVM development process while using only a single subject's data. Further individualization was conducted in the feature selection process, to examine the difference in the most important feature contributions to each subject's individualized model. The feature importance graph (Figure 4) highlights how features were selected to further individualize each subject's model. After creating the classifier using the entire feature set, the top 10 features were selected from the output (all features above the black dashed line in Figure 4) and were included as the only features in a new classifier built specifically for that subject. This process resulted in 3 different feature sets: all features, Top 10 features, and classic CWL metrics. Tables 8, 9 present the accuracy metrics for each model across each subject, including the combination of all subjects to serve as a generalized model comparison.

**Figure 5:**
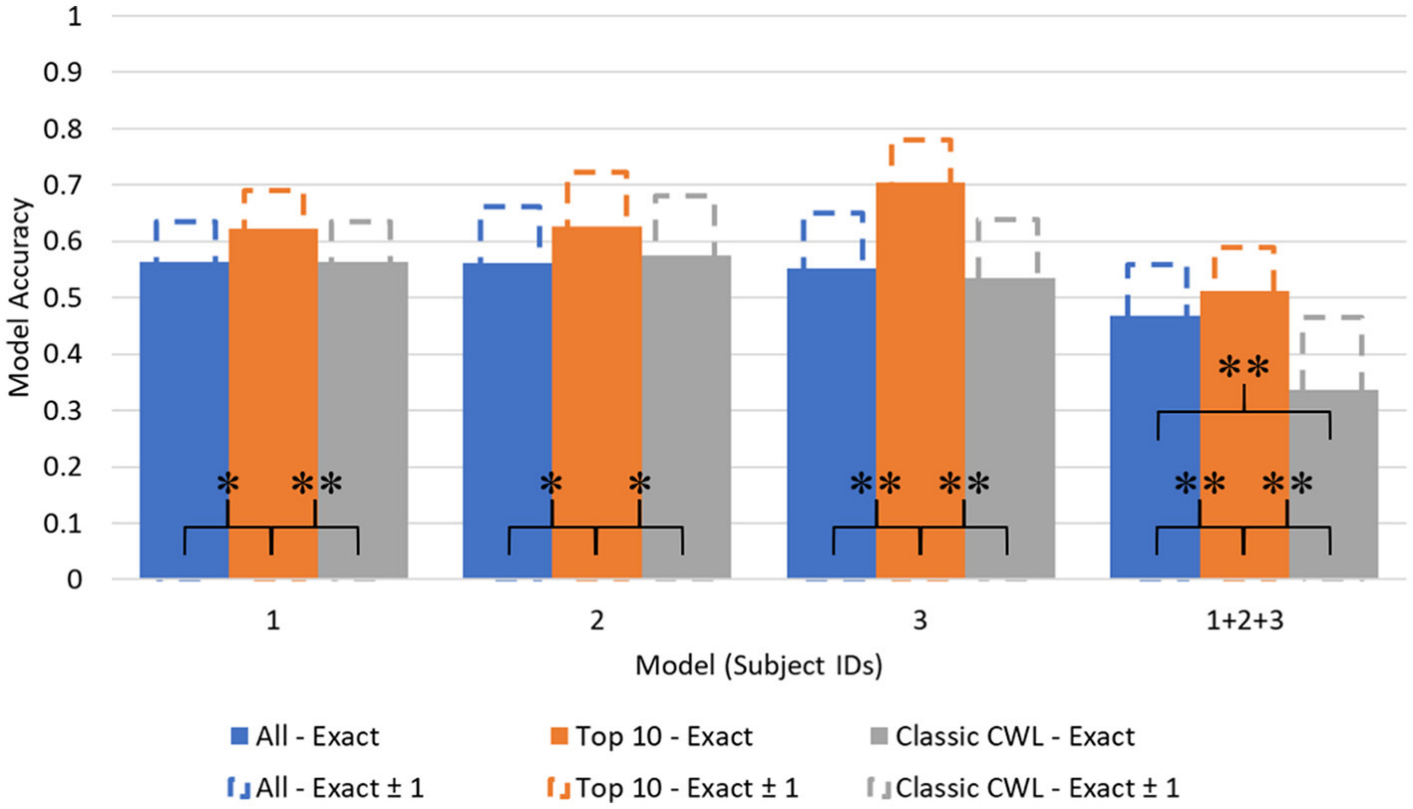
Demand level prediction accuracy across models. *p < 0.01, **p < 0.001.

**Figure 6:**
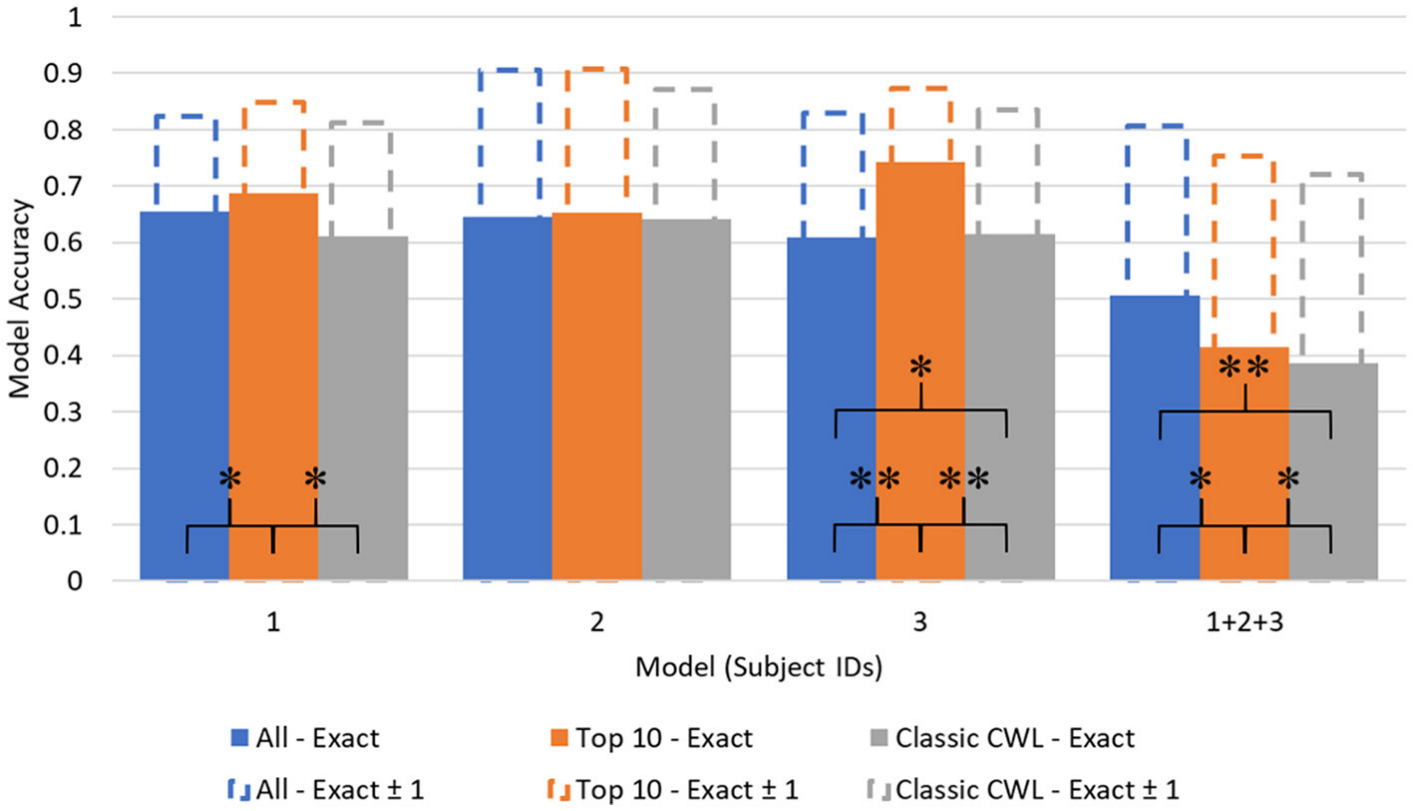
CSS prediction accuracy across models. *p < 0.01, **p < 0.001.

Figures 5, 6 depict the accuracy values across each model. Additionally, another metric labeled as Accuracy (Exact ± 1) is depicted as extensions of each accuracy bar. The Accuracy (Exact ± 1) metric represents how accurate the model is when its predictions are close to the true value. To derive the Accuracy (Exact ± 1) metric, the diagonal of the confusion matrices is summed with the neighboring prediction proportions. For example, in Table 6, the true positive value of demand level 5 is the sum of prediction 4, 5, and 6 proportions in the True 5 row. Of course, this artificially heightens the accuracy value but leads to potentially interesting discussion points relevant to safety-critical domains.

No significant deviations in normality were found among the cross validated accuracy distributions using a Shapiro-Wilk test (W = 0.775, *p* = 0.05, for the most offending distribution set), so a parametric test was chosen for model comparison. The differences between accuracy values as a function of feature set were evaluated using Student's paired *t*-tests to identify where statistical differences in model performance occurred. The results of the statistical model comparisons are included in Figures 5, 6, represented as linked comparisons significant at *p-*values < 0.01 (^*^) and < 0.001 (^**^).

Significant differences between model accuracy scores were found between feature sets consistently within the demand level classifier. The Top 10 feature models performed significantly higher than the complete and CWL metric feature sets, with an average increase in accuracy equal to 0.1003 over the other feature sets (All: 0.5361; Top 10: 0.6165; CWL: 0.5022). Another significant difference was observed between the complete feature set (*M* = 0.4590, *SD* = 0.0083) and the CWL metric feature set (*M* = 0.3756, *SD* = 0.0099) in the combined subject model, *t*_(4)_ = 14.6, *p* < 0.001. The complete feature set performed significantly better than the CWL feature set, with an average difference in accuracy equal to 0.0834. Individual subject models of demand level prediction demonstrated no significant differences between the entire feature set and the reduced CWL metric feature set, indicating that the reduction in model complexity did not significantly alter the model's predictive accuracy.

The CSS classifier accuracy values also showed significant differences between feature sets. No significant differences were found across feature sets for subject 2's models, even when individualized to their data and top features. Again, the Top 10 feature set performed at higher levels of predictive accuracy compared to the complete or CWL metric feature sets for subjects 1 and 2. An average of 0.0826 difference in accuracy scores persisted between these feature sets (All: 0.6316; Top 10: 0.7158; CWL: 0.6349). A small, but statistically significant, difference also occurred between the complete feature set (*M* = 0.5883, *SD* = 0.0090) and CWL metric feature set (*M* = 0.6222, *SD* = 0.0146) in subject 3's individualized models, *t*_(4)_ = −5.05, *p* = 0.007. The CWL metric feature set provided an increase of accuracy equal to 0.0339 relative to the complete feature set, indicating that the model performed better with less input data. In the combined subject CSS classifiers, the complete feature model performed significantly better than the Top 10, *t*_(4)_ = 6.12, *p* = 0.004, and CWL metric, *t*_(4)_ = 9.13, *p* < 0.001, feature sets, with an average increase of 0.1062 in accuracy. In fact, each step down in feature set complexity yielded a statistically significant reduction in the combined subject model accuracy [*t*_(4)_ = 4.64, *p* = 0.010: between the Top 10 and CWL metric feature set].

**Figure 7:**
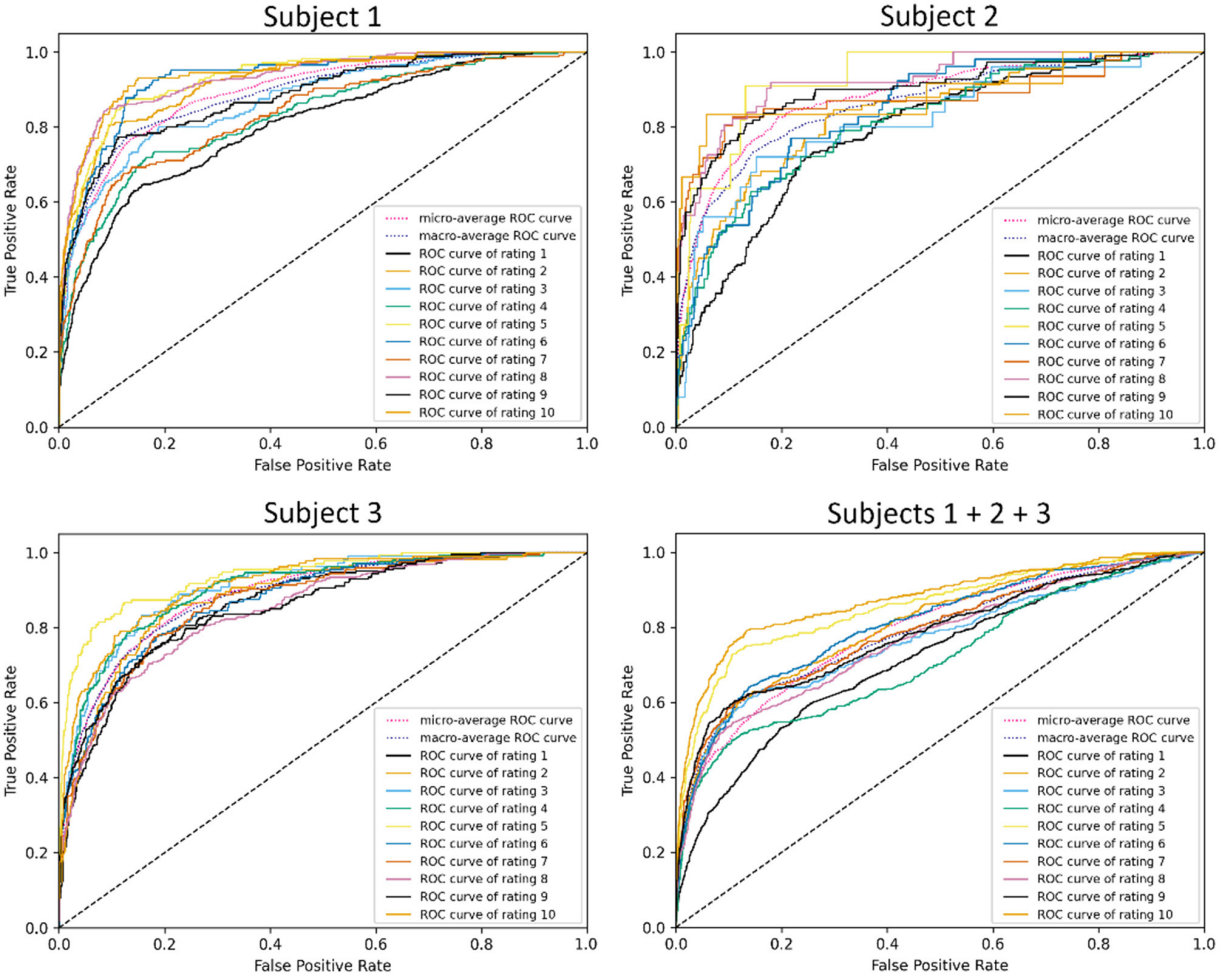
ROC visualizations for demand level classifiers built using all features across all subjects.

**Figure 8:**
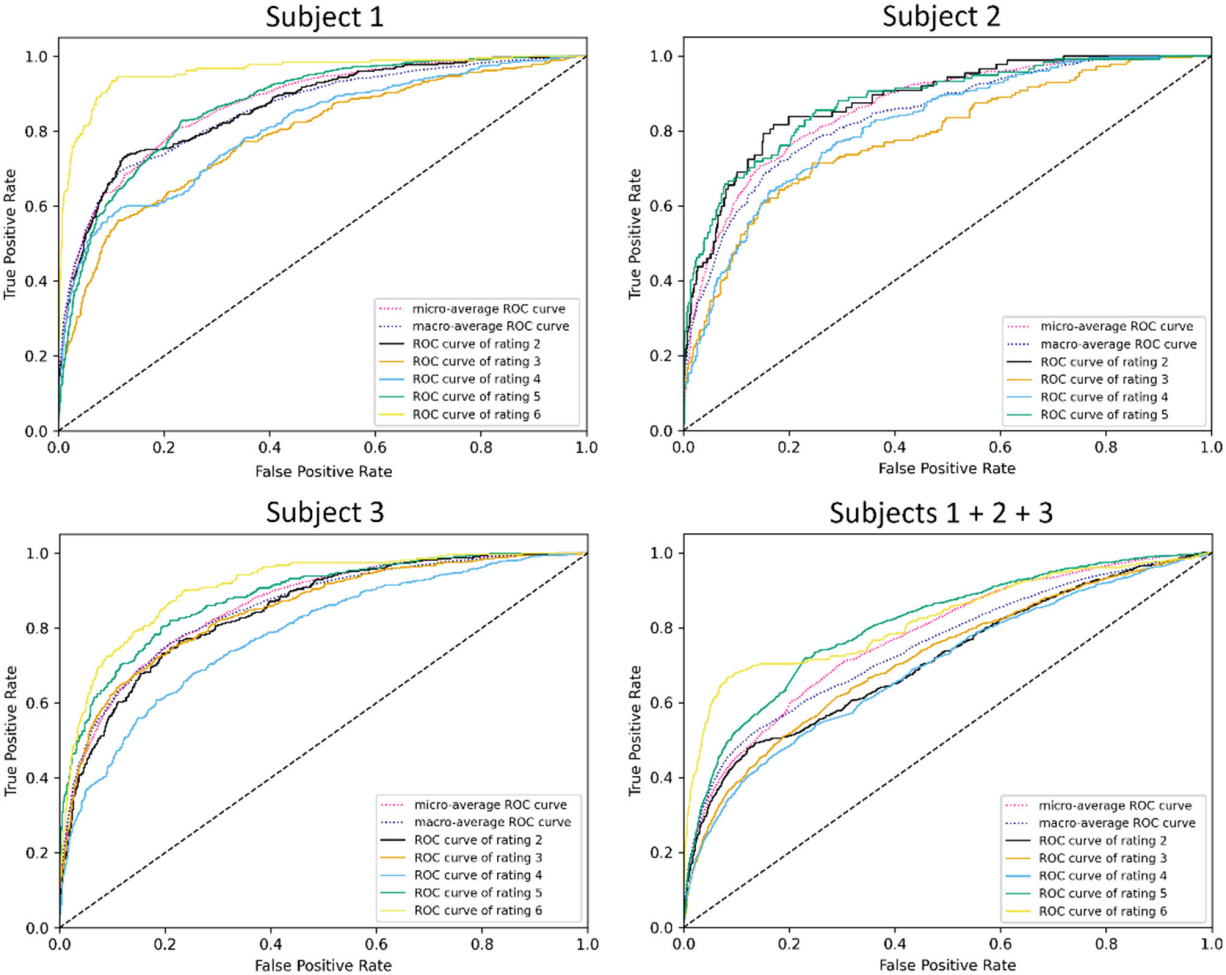
ROC visualizations for CSS classifiers built using all features across all subjects.

ROC visualizations are included (Figures 7, 8) for each classifier (demand level and CSS, respectively) using the complete feature set, showing curves for each individual class as well as microaverage and macroaverage curves across classifications. As visible in Figures 7, 8, differences in predictive performance occur between the multiclass structure of the models. To explore this further, recall, precision, and AUC-ROC values were calculated for each individual class to better understand the nature and potential causes of these differences. Table 10 provides these metrics for each corresponding ROC curve, providing a measure of predictive accuracy for each class.

**Table 10:** Individual class recall, precision, and AUC-ROC values.

**Subject**	**Demand level classifier**				**CSS classifier**			
	**1**	**2**	**3**	**1**+**2**+**3**	**1**	**2**	**3**	**1**+**2**+**3**
Macroaverage Recall	0.535	0.461	0.5065	0.4458	0.6128	0.5658	0.604	0.4681
Macroaverage Precision	0.5945	0.5349	0.5391	0.5052	0.6636	0.6678	0.6062	0.5507
Macroaverage AUC-ROC	0.89	0.86	0.88	0.79	0.86	0.84	0.85	0.75
Microaverage AUC-ROC	0.89	0.88	0.89	0.79	0.87	0.86	0.86	0.77
AUC-ROC 1	0.8	0.79	0.86	0.71	N/A	N/A	N/A	N/A
AUC-ROC 2	0.92	0.84	0.88	0.81	0.86	0.88	0.85	0.71
AUC-ROC 3	0.87	0.82	0.91	0.77	0.79	0.78	0.85	0.72
AUC-ROC 4	0.83	0.82	0.9	0.72	0.81	0.81	0.78	0.69
AUC-ROC 5	0.93	0.93	0.94	0.86	0.87	0.88	0.88	0.81
AUC-ROC 6	0.93	0.84	0.87	0.81	0.96	N/A	0.91	0.82
AUC-ROC 7	0.83	0.88	0.87	0.79	N/A	N/A	N/A	N/A
AUC-ROC 8	0.93	0.93	0.85	0.77				
AUC-ROC 9	0.88	0.9	0.86	0.78				
AUC-ROC 10	0.94	0.89	0.91	0.88				
Precision 1	0.4622	0.5376	0.5018	0.3591	N/A	N/A	N/A	N/A
Precision 2	0.694	0.5250	0.4713	0.4943	0.6006	0.7692	0.5341	0.6173
Precision 3	0.5427	0.3000	0.5165	0.4418	0.6316	0.5886	0.6252	0.5020
Precision 4	0.5068	0.5375	0.5763	0.4913	0.6496	0.6504	0.6161	0.4240
Precision 5	0.6370	0.3333	0.7054	0.5663	0.6629	0.6667	0.6304	0.6064
Precision 6	0.5785	0.3469	0.5549	0.5016	0.7733	N/A	0.625	0.6039
Precision 7	0.6045	0.5745	0.5488	0.5324	N/A	N/A	N/A	N/A
Precision 8	0.7171	0.7143	0.5269	0.5606				
Precision 9	0.6266	0.73	0.5510	0.5381				
Precision 10	0.5755	0.75	0.6478	0.5663				
Recall 1	0.7032	0.7042	0.5417	0.6078	N/A	N/A	N/A	N/A
Recall 2	0.5812	0.4615	0.4302	0.4513	0.5395	0.3448	0.5555	0.2170
Recall 3	0.5057	0.1200	0.4352	0.3245	0.4635	0.5110	0.6843	0.4276
Recall 4	0.4458	0.5000	0.6214	0.4072	0.5640	0.8261	0.5826	0.6766
Recall 5	0.5284	0.1818	0.6741	0.4393	0.8629	0.5812	0.5932	0.5846
Recall 6	0.4762	0.3269	0.5050	0.3703	0.6339	N/A	0.6044	0.4346
Recall 7	0.3553	0.5870	0.4592	0.4266	N/A	N/A	N/A	N/A
Recall 8	0.6631	0.5645	0.6481	0.4892				
Recall 9	0.5351	0.6636	0.5070	0.4388				
Recall 10	0.5556	0.5000	0.5691	0.5029		All		

Values are relatively (i.e., within the same metric) color coded as a heat map (blue = high, red = low) for easier viewing.

The cells in Table 10 are highlighted as a heatmap to demonstrate where high and low values of the accuracy metrics are distributed throughout the individual class classifiers. This emergent feature demonstrates the trend of the combined subject model having a general reduction in recall, precision, and AUC-ROC. It is also interesting to note that the lower values propagate more toward lower-level classes that are more representative of low CWL conditions. Lastly, the balance between the micro and macroaverage values in the AUC-ROC scores suggests that models perform similarly well across all classes. No single class disproportionately skews the overall performance, and there are likely no classes with extremely poor or exceptionally high discrimination, relatively speaking.

## 4 Discussion

Aviation has consistently been at the forefront of CWL research due to its high-stakes environment, where variability in cognitive demand can lead to critical errors with devastating consequences. This study demonstrates that even brief periods of physiological data can predict task demands and subjective workload ratings with surprising accuracy. Unlike traditional methods that rely heavily on subjective self-report or limited objective metrics, the models in this work leveraged machine learning to process physiological signals and provide granular workload predictions. Across accuracy, precision, recall, AUC-ROC, and MCC metrics, the models demonstrated high predictive capacity, comparable to or exceeding prior literature employing binary or trinary CWL ratings (Hart and Staveland, 1988; Gevins and Smith, 2003). Furthermore, SVMs offer a fully auditable production pipeline, providing transparency in the decision-making process—a key advantage over more opaque deep learning methods that dominate the current literature and a necessity in safety-critical or otherwise regulated applications.

The study's first aim focused on developing binary classifiers as a baseline to evaluate the potential of physiological data for workload prediction. The binary classifiers demonstrated strong performance, with AUC-ROC values of 0.77 for demand level and 0.84 for CSS ratings using all features. These metrics reflect the models' capacity for fair to good discrimination, particularly in distinguishing between high and low workload conditions. Interestingly, the binary CSS classifier consistently outperformed the demand level classifier across all metrics, including MCC values (0.4840 vs. 0.4097), which further supports prior findings that subjective CWL ratings often exhibit stronger correlations with physiological data than objective task demands (Cain, 2007; Hart and Staveland, 1988). The CSS classifier's superior performance underscores its potential for real-time operational monitoring, where subjective experiences may better reflect cognitive strain than external task metrics that may be more difficult to access and assess accurately.

Feature selection emerged as a critical determinant of model performance. For binary classifiers, the All Features model demonstrated the strongest overall performance, achieving an exact accuracy of 0.7052 for demand level predictions and 0.8042 for CSS ratings. However, the Top 10 Features model provided a compelling alternative, achieving comparable performance while reducing the dimensionality of the input data. For example, the binary CSS classifier achieved an AUC-ROC of 0.83 and an MCC of 0.5230 with the Top 10 feature set, suggesting that these features retained the most critical physiological indicators of workload. In contrast, the Classic CWL Metrics model consistently underperformed, with AUC-ROC values dropping to 0.64 for demand level and 0.75 for CSS. This finding aligns with literature emphasizing the importance of tailored feature selection in optimizing model performance (Guyon et al., 2002). By reducing model complexity while maintaining robust predictive power, the Top 10 Features model offers a practical balance between interpretability and accuracy, particularly for applications requiring efficient processing.

Across all models, descriptive pupil diameter metrics, such as mean, minimum, and maximum values, consistently demonstrated high levels of feature importance. Of the ECG metrics collected, the mean heart rate had consistently high levels of feature importance for each model. This contrasts with the heart rate variability metrics that were *a priori* expected to be influential features in the model development. As such, two out of the five classic CWL metric feature set were inconsistent with past literature where the metrics did yield success in identifying CWL changes. This difference may be due to the transient nature of the two physiological metrics being captured in this study. Pupil diameter changes operate across short time intervals, reacting dynamically to changes in the environment. Heart rate variability, however, is less dynamic and may demonstrate more consistency over short intervals, such as the small ~12 s sliding window and 5-min trials employed in the current study. Longer task durations or across-session analysis may provide better insight to the value of heart rate variability in the context of its inclusion in future iterations of these models.

Building on the binary classifiers, the study's second aim expanded the scope to multiclass classification, offering greater granularity in CWL predictions. While binary models dichotomize CWL into high and low states, multiclass models provide finer resolution, capturing CWL levels along a spectrum. In the context of the region model, the binary classification models provide a classification between region A2 (optimal CWL) and region B (overloaded CWL) with region A3 (increasing CWL) fuzzily split between the two groups. The multiclass classifiers provide finer resolution to the fuzzy split of region A3, providing classifications along the continuum of increasing CWL up to the red line boundary between regions A3 and B. This build up to the red line is particularly valuable in real-world applications, where early remedial actions to reduce CWL can have significant operational implications. For example, in aviation, identifying the progression from moderate to high CWL could enable adaptive automation systems to intervene before overload occurs, improving safety and efficiency (Wickens and Hollands, 2000). Similarly, in healthcare, detecting incremental increases in CWL could help clinicians manage cognitive fatigue during complex procedures (Gevins and Smith, 2003).

Despite the increased complexity, the multiclass models demonstrated reasonable performance relative to their binary counterparts. The combined-subject demand level classifier achieved an AUC-ROC of 0.79, while the CSS classifier reached 0.75. These values indicate fair discrimination, albeit lower than the binary models, which achieved AUC-ROC values of 0.77 and 0.84, respectively. The Matthews Correlation Coefficient (MCC) further highlighted this trade-off, with the Top 10 Features model achieving MCC values of 0.4499 for demand level and 0.3553 for CSS in the multiclass setting, compared to 0.4641 and 0.5230 for their binary equivalents. These findings suggest that while multiclass classification sacrifices some predictive accuracy, it provides valuable granularity that can enhance the interpretability and applicability of workload predictions in dynamic environments.

Class-specific analysis revealed important insights into the challenges of multiclass classification. Both demand level and CSS classifiers struggled with low and moderate CWL conditions, as evidenced by lower precision and recall values for classes representing these states. For example, the demand level classifier's AUC-ROC for classes 1–3 averaged approximately 0.71 in the combined model, while precision and recall for class 3 were notably low (e.g., precision: 0.5020, recall: 0.4276). These results suggest that physiological signatures for low and moderate CWL states are less distinct, leading to greater overlap in feature spaces and reduced separability. Conversely, higher CWL conditions consistently achieved the highest performance metrics, likely reflecting the stronger physiological responses associated with cognitive overload, such as increased heart rate and pupil dilation (Wickens and Hollands, 2000; Chen and Epps, 2013). These trends align with the resource supply hypothesis, which posits that physiological changes become more pronounced as individuals approach cognitive capacity limits.

The third aim of this study explored the potential for individualized models to enhance multiclass classification performance. Combined-subject models, while valuable for generalization, inherently face challenges in accommodating inter-subject variability. Physiological responses to CWL are highly individual, influenced by factors such as baseline cognitive capacity, stress tolerance, and task familiarity (Cain, 2007; Fairclough, 2009). By tailoring models to individual subjects, it is possible to capture these unique patterns, resulting in improved predictive performance. For example, Subject 3's individualized multiclass demand level classifier achieved an AUC-ROC of 0.95 and an MCC of 0.6674 using the Top 10 Features, far surpassing the performance of the combined model. Similarly, the individualized CSS classifier achieved an AUC-ROC of 0.93 and an MCC of 0.6712, reflecting its exceptional ability to predict nuanced CWL ratings.

Feature selection also emerged as a key consideration in the development of individualized models. The Top 10 Features consistently outperformed both the All Features and Classic CWL Metrics models, emphasizing the importance of identifying the most informative physiological indicators. Metrics such as mean heart rate and pupil diameter ranked highly across subjects, while heart rate variability unexpectedly showed less consistent contributions. These findings align with prior research suggesting that streamlined feature sets can enhance both interpretability and performance by reducing noise and redundancy in the data (Guyon et al., 2002; Fawcett, 2006).

### 4.1 Limitations

It is important to note that the low sample size utilized in the current study may affect the generalizability of the combined subject model presented in this work. While individualized CWL predictive models demonstrated increased accuracy, a larger number of subjects would allow for a more robust combined subject model and solidify the necessity of individualized models if they all demonstrate significantly increased predictive accuracy relative to the combined subject model. Additional benefits include improving the robustness of the dataset in terms of class distributions to avoid any imbalanced class issues.

Data collected in this study was collected in a laboratory environment, eliminating operational factors, such as environmental factors and confounding autonomic states (such as emotional responses to high-risk scenarios or high levels of fatigue). Replicating the study in a high-fidelity aviation simulator would serve to determine if the same predictive accuracy can be achieved under more realistic operational conditions. The USAARL MATB may be aviation-like, but further work is needed to identify if higher-fidelity operationally relevant tasks would yield similar results across a larger sample. Additionally, military aviators are likely to be more homogenous in physiological responses than the general populace, especially when performing cognitively demanding aviation-like tasks. This homogeneity may lend itself to improved combined subject model performance.

### 4.2 Study implications and future work

This demonstration, which is reproducible through the approach outlined in this document, has two near-term applications which may radically accelerate future aviation development. First, accurately estimating cognitive workload from physiologically derived features provides the basis for OSM and true adaptive automation to expand the operational capabilities of current and future pilots while decreasing risk of the hazards and stresses associated with cognitive underload and overload. Using a sliding window as short as 12 s allows for continuous prediction of CWL throughout the performance of a task. It is theoretically possible to create an adaptive automation system that leverages this continuous predictive stream to initiate remedial actions (such as turning on assistive automation systems) when CWL predictions pass a specific threshold. The resulting model and methodology produced in this work supports these future projects.

Second, rapid, accurate, non-intrusive estimation of CWL can substantially reduce the time needed between iterative tests in rapid prototyping of cognitively demanding systems. By employing a physiologically driven CWL assessment model, an objective assessment of CWL can be derived to aid in the comparisons between the configurations of tested protypes under evaluation. Systems with lower CWL predictions would be indicative of increased usability and acceptance of the system. As additional features and functionality are added to a system, objective changes in CWL can be monitored through physiological means, which are less likely to be affected by top-down factors such as cognitive bias or emotion.

In future iterations of this modeling process, additional physiological features and/or contextual factors should be used to bolster the predictive accuracy of the model. Other physiological measures have demonstrated the ability to serve as proxy measures for CWL assessment, such as electroencephalogram (EEG; Zhou et al., 2021), functional near infrared spectroscopy (fNIRS; Aghajani et al., 2017), and electrodermal activity (EDA; Braithwaite et al., 2013), to name a few examples. The CWL metrics derived from these sensors should be incorporated into this SVM as additional features used to make its prediction. From this, continued feature selection methodologies including dimensionality reduction methods such as principal component analysis (PCA), can be incorporated into future model refinement. Additionally, contextual factors can provide valuable weighting to situations known to be more cognitively complex compared to other scenarios that are less demanding.

## 5 Conclusions

In conclusion, this study highlights the potential of SVM classifiers for CWL prediction, emphasizing the trade-offs between binary and multiclass classification, combined and individualized models, and comprehensive versus streamlined feature sets. While binary classifiers exceled in their simplicity and reliability, multiclass models provided valuable granularity that can enhance the interpretability and applicability of CWL predictions when using physiological measures that are sensitive to CWL changes in Regions A1 and A3. The superior performance of individualized models emphasizes the importance of tailoring classifiers to specific physiological patterns, particularly in applications where accuracy and reliability are paramount. These findings provide a foundation for future research aimed at developing adaptive and scalable CWL prediction systems, with implications for improving safety, efficiency, and performance in high-stakes environments. In summary:

1. We present models that accurately predict the USAARL MATB task demand and CSS subjective scores based on objective measures of ECG and eye tracking. Binary classifiers for both demand level and CSS achieved high discriminatory power, with accuracy values of 70 and 81%, respectively, when using optimized feature sets. Multiclass model performance for USAARL MATB demand level exceeded 70% accuracy for demand level and CSS predictions exceeded 74% accuracy in the most optimized models.
2. The expected results of the SVM generalized models were random chance performance: 10% for predicting 10 levels of the USAARL MATB and 20% for the 5 ratings of the CSS used here. The resulting multiclass models presented here exceeded *a priori* expectations in results by a large margin and held its performance against traditional binary classifiers.
3. The accuracy of these multiclass models suggests that physiology alone can be used to identify level of CWL required across a finer resolution of task demands resulting in differing subjective ratings.
4. Subjective workload classifiers outperformed demand level classifiers across most metrics, suggesting subjective workload ratings may correlate more directly with physiological data than objective task demands.
5. We demonstrate increased predicative accuracy (an average of +13.9% for task demand and +13.0% for CSS rating predictions) for individualized multiclass models compared to generalized models (when comparing the highest accuracy feature sets). Personalization captured subject-specific physiological responses, highlighting its critical role in applications where high accuracy is essential, such as aviation and healthcare.
6. The Top 10 feature set consistently outperformed the Classic CWL metrics and provided competitive results compared to the All Features set. Streamlined feature sets enhanced computational efficiency while retaining strong predictive performance, emphasizing the importance of feature engineering in workload models.
7. Binary classifiers are well-suited for tasks requiring simplicity and efficiency, while multiclass models excel in dynamic environments where granular workload assessments provide added value. Tailoring model selection to the specific operational context, such as adaptive automation or clinician support, can enhance safety, performance, and decision-making.

## Data Availability

The raw data supporting the conclusions of this article will be made available by the authors, without undue reservation.
